# Cross-generational genomic prediction of Norway spruce (*Picea abies*) wood properties: an evaluation using independent validation

**DOI:** 10.1186/s12864-025-11861-x

**Published:** 2025-07-21

**Authors:** Haleh Hayatgheibi, Henrik R. Hallingbäck, Salvador A. Gezan, Sven-Olof Lundqvist, Thomas Grahn, Gerhard Scheepers, Sonali Sachin Ranade, Katri Kärkkäinen, M. Rosario García Gil

**Affiliations:** 1 Department of Forest Genetics and Plant Physiology, Swedish University of Agricultural Sciences (SLU), Umeå, Sweden; 2 Forestry research Institute Sweden (Skogforsk), Uppsala, 75183 Sweden; 3 VSN International Ltd, Hemel Hempstead, UK; 4 IIC, Rosenlundsgatan 48B, Stockholm, 118 63 Sweden; 5 RISE Research Institutes of Sweden AB (RISE), Stockholm, 114 86 Sweden; 6 Natural Resources Institute Finland (LUKE), Oulu, Finland

**Keywords:** Cross-generation, Genomic selection, GBLUP, Norway Spruce, Cambial age, Wood properties

## Abstract

**Background:**

The evaluation of genomic selection (GS) efficiency in forestry has primarily relied on cross-validation schemes that split the same population within a single generation for both training and validation. While useful, this approach may not be reliable for multigenerational breeding. To our knowledge, this is the first study to assess genomic prediction in Norway spruce using a large dataset spanning two generations in two environments. We trained pedigree-based (ABLUP) and marker-based (GBLUP) prediction models under three approaches: forward prediction, backward prediction, and across-environment prediction. The models were evaluated for ring-width, solid-wood and tracheid characteristics, using ~ 6,000 phenotyped and ~ 2,500 genotyped individual. Predictive ability (PA) and prediction accuracy (ACC) were estimated using an independent validation method, ensuring no individuals were shared between training and validation datasets. To assess the trade-off between comprehensive radial history and practical direct methods, we compared GBLUP models trained with cumulative area-weighted density (AWE-GBLUP) and single annual-ring density (SAD-GBLUP) from mother plus-trees. These models were validated using early and mature-stage progeny density measurements across two trials.

**Results:**

Despite the smaller number of individuals used in the GBLUP models, both PA and ACC were generally comparable to those of the ABLUP model, particularly for cross-environment predictions. Overall, forward and backward predictions were significantly higher for density-related and tracheid properties, suggesting that across-generation predictions are feasible for wood properties but may be challenging for growth and low-heritability traits. Notably, SAD-GBLUP provided comparable prediction accuracies to AWE-GBLUP, supporting the use of more practical and cost-effective phenotyping methods in operational breeding programs.

**Conclusions:**

Our findings highlight the need for context-specific models to improve the accuracy and reliability of genomic prediction in forest tree breeding. Future efforts might aim to expand training populations, incorporate non-additive genetic effects, and validate model performance across cambial ages while accounting for climatic variability during the corresponding growth years. Overall, this study offers a valuable foundation for implementing GS in Norway spruce breeding programs.

**Supplementary Information:**

The online version contains supplementary material available at 10.1186/s12864-025-11861-x.

## Background

The concept of using genome-wide DNA markers to predict genetic merit of individuals, subsequently called “genomic selection” (GS), has revolutionized animal and plant breeding in the last two decades [1, 2]. In essence, GS is a form of marker-assisted selection (MAS) that utilizes marker-trait associations [3]. Nevertheless, unlike MAS, which uses a few markers linked to large-effect quantitative trait loci (QTLs), GS does not necessarily require prior identification of the associations between phenotypes and markers, the genomic locations of QTLs, or their relative effects on the phenotype [4]. Principally, GS models are trained on an available set of phenotypic and marker data from a training set (TS) to establish a statistical model that predicts breeding values of genotyped but non-phenotyped individuals, referred to as selection candidates or the validation set (VS) [5, 6].

Genomic prediction models, coupled with drastically reduced genotyping costs, have led to significant gains in key crop and livestock traits while shortening the time needed for conducting well-informed selection [7]. In forest tree breeding, conventional breeding methods have consistently improved traits such as, tree volume [8], wood quality [9, 10] and stem straightness [11]. However, forest tree breeding is still in its early stages, hindered by long breeding cycles, delayed and poor flowering, and late expression of economically important traits [2]. For instance, tree improvement of boreal conifer species typically involves breeding cycles that exceeds 20 years or more, including creation of crosses, field evaluation of progenies, selection of superior individuals, and the subsequent propagation of selected material through either sexual or vegetative methods [12]. This time constraint along with the high cost of field evaluations underscores that the impact of GS on forest tree breeding could be even greater than in agricultural crops or animal breeding programs [2]. Additionally, what distinguishes GS from traditional breeding methods is its use of DNA data to construct the realized relationship matrix between individuals (G-matrix) rather than relying only on pedigree-based relationship matrix (A-matrix), which is often prone to errors [13, 14]. This advancement, not only enhances pedigree accuracy, but may also capture within-family variation resulting from random Mendelian segregation [14, 15].

Nevertheless, the performance of GS is determined by its accuracy, which reflects its ability to predict a breeding value for trees that lack phenotypic data. Overall, prediction accuracy (ACC) is a trait- and population-specific parameter and is influenced by several factors, including the heritability of the target trait, the size of TS, the degree of genetic relatedness between the TS and VS, and the extent of linkage disequilibrium (LD) between markers and QTLs. The degree to which LD can be leveraged for improving predictions is determined by marker density and effective population size ($\:{N}_{e}$) [16, 17].

Since the first GS studies in forest trees [18, 19], it has become evident that ACC is highest when models are applied to related trees of the same age and grown under the same environmental conditions similar to those of the TS. Numerous studies in conifers [20–22] and eucalyptus [23] have confirmed the importance of genetic relationships, as well as genotype-by-environment interaction (G×E) and age-age correlations. These findings align with similar observations in domestic animals and crops [24]. However, while high predictive ability (PA) within the same cohort and environment is often observed, it is not necessarily the primary goal in operational breeding, where selection across different environments, generations, or ages is typically required.

A common method for evaluating GS model performance is k-fold cross-validation, where individual observations are randomly divided into k subsets. All but one subset is used as the TS, while the remaining subset serves as the VS, with its phenotypes set to missing. The correlation between predicted and observed phenotypes across multiple iterations is used to measure the PA [25]. Although this approach is useful, the results often shows a misleadingly optimistic view of GS potential because the same population is used for both model development and validation [26]. Among other limitations, this method does not account for changes in the marker–trait linkage phase, which may lead to an overestimation of model accuracy. To more accurately assess the efficiency of GS in forest trees, the population must undergo breeding to observe the effects of recombination on the marker-trait phase. In other words, a model developed for the current generation should be validated independently in a subsequent generation and/or tested in a separate environment for a more robust evaluation [14, 27].

In general, G×E interaction is low for wood properties [28]. A previous study using the same trial sites in the current study also reported low G×E for various wood traits, based on phenotypic data collected from these sites [29]. Similarly, The accuracy of GS for tree height and wood quality traits in Norway spruce (*Picea abies* L.) has previously been evaluated within a single generation using a cross-validation design [22, 30, 31]. However, due to the ongoing advancement of GS programs for Norway spruce and the significant changes in wood chemical and physical properties from the juvenile to mature phases of secondary growth, it is essential to retrain and test GS models across generations, environments, and developmental stages to ensure that predictions remain aligned with those from earlier stages.

In this study, we conducted genomic prediction for economically important wood traits in Norway spruce using genotypic and phenotypic data from two generations across two half-sib progeny trials (Höreda and Erikstorp) and their parent clones located in breeding archives in Sweden. The specific objectives were:

(i) To assess narrow-sense heritability ($\:{h}^{2}$), PA, and ACC of pedigree-based models (ABLUP) and marker-based GS models (GBLUP), we evaluated three different approaches involving training and validation across environments—and in most cases, across generations. Approach A: training the models on the phenotypic data of the parental generation (G0), which consists of plus trees—that is, individuals with superior phenotypes selected from natural stands based on traits of economic or ecological importance, and validating predictions for progenies (G1) in Höreda (G1H) and Erikstorp (G1E), Approach B: training the models on the phenotypic data from G1H and validating predictions for G1E and G0 trees, and Approach C: training the models on the phenotypic data from G1E and validating predictions for G1H and G0 trees; (ii) To assess the efficiency of early training in GBLUP for wood density and to evaluate whether wood juvenility influences the outcomes of selection, under Approach A and; (iii) To evaluate the impact of different measurement methods [accumulated area-weighted wood density (AWE) versus single annual-ring direct density (SAD)] on the efficiency of GBLUP model under Approach-A. This comparison is motivated by practical considerations, as AWE provides a more integrated measure over time but is more labour-intensive, whereas SAD may offer a quicker, lower-cost alternative for operational breeding.

## Methods

### Plant material

The Norway spruce breeding program in Sweden began in the 1940 s with the phenotypic selection of approximately 900 plus trees (the G0 population) from across most of the species’ range [32], followed by the establishment of the first round of seed orchards [33]. Progeny (G1) from the G0 trees were obtained either through controlled crosses to generate full-sib progenies or through open-pollination, resulting in half-sib progenies. Progeny testing of the plus trees began in 1971, 30 years after their selection, to develop the next generation population (G1).

In the 1980 s, additional plus trees selected from commercial forest nurseries, together with selection of the best progeny-tested plus trees from the previous selection resulted in the second round of seed orchards. The third round of seed orchards were established in 21 st century by genetic testing of the progenies of plus trees.

This study utilized phenotypic and, where available, genomic data from a two-generation Norway spruce pedigree. The maternal generation consists of about 1,300 plus tree clones, from which two large open-pollinated progeny trials, Höreda (S21F9021146) (57.61°N, 15.04°E) and Erikstorp (S21F9021147) (55.90°N, 13.93°E), were established by the Forestry Research Institute of Sweden (Skogforsk) in southern Sweden in 1990. The experimental design for each trial follows a randomized incomplete block layout, utilizing single-tree plots. The Höreda trial comprises 1,373 half-sib families distributed across 20 blocks, while the Erikstorp trial includes 1,375 half-sib families divided into 23 blocks [29].

## Phenotypic and genotypic data

In 2010 and 2011, two 12-mm bark-to-pith increment cores were collected at a height of 1.3 m from progenies at ages 20 to 21 years old, respectively. These progenies originated from 524 out of 1,300 G0 mother plus trees established as grafts in various breeding archives in southernmost Sweden in 1986. A total of six progenies were sampled per family and per progeny trial, Höreda and Erikstorp, resulting in about 6,000 progenies, which were phenotyped for high-resolution data on pith-to-bark radial variations in different solid wood and tracheid properties, using the SilviScan instrument at Innventia (now RISE) in Stockholm, Sweden. From these variations, growth rings and their segments of earlywood (EW), transition wood (TW), and latewood (LW) were identified, and the widths and mean wood and tracheid properties were calculated for all rings and segments [34]. Additionally, a 12-mm bark-to-pith increment core was collected from one ramet (a clonally propagated copy) from each of the 524 G0 mother plus trees and analysed using SilviScan instrument.

In this study, we primarily focused on wood density in growth rings (DENS) and their three ring segments: earlywood density (EWDENS), transition wood density (TWDENS), and latewood density (LWDENS). We also assessed ring width (RWT), representing secondary tree growth, along with modulus of elasticity (MOE), microfibril angle (MFA), tracheid wall thickness (TWTH), and tracheid coarseness (TC). In summary, these wood properties were measured in 524 presumably unrelated mother plus-trees (G0 trees), 3,788 G1 open-pollinated offspring trees from Höreda (G1H), and 2,664 G1 trees from Erikstorp (G1E).

Because area-weighted values (AWE) more accurately represent the average properties of the wood [35], the AWE for each trait was calculated and used in this study as follows:

1 $$\:AWE=\frac{\sum\:\left({a}_{i}{d}_{i}\right)}{\sum\:\left({a}_{i}\right)}$$

where $\:i$ is the cross-sectional area of annual ring $\:i$, assuming that each ring is circular, and $\:{d}_{i}$is the value of annual ring $\:i$.

Furthermore, genotypic data were used for 518 G0 trees, 1,684 G1 trees from Höreda originating from 1,339 half-sib families (with 1,274 of families represented by only one individual per family and 65 families represented by approximately 6.2 individuals per family). Additionally, 303 G1 trees from Erikstorp originating from 65 half-sib families (represented by an average of 4.6 individuals per family) were genotyped.

The number of families subjected to genotyping was much higher in the Höreda trial compared to the Erikstorp trial due to sampling strategies used in earlier studies. Initially, 404 individuals from 65 families in Höreda and 303 individuals from similar families in Erikstorp were sampled for GS studies [30]. Later, for genome-wide association studies (GWAS), which required genetically unrelated individuals, an additional 1,280 individuals from Höreda were sampled, with only one individual representing each family.

## DNA extraction and genomic data

**Fig. 1:**
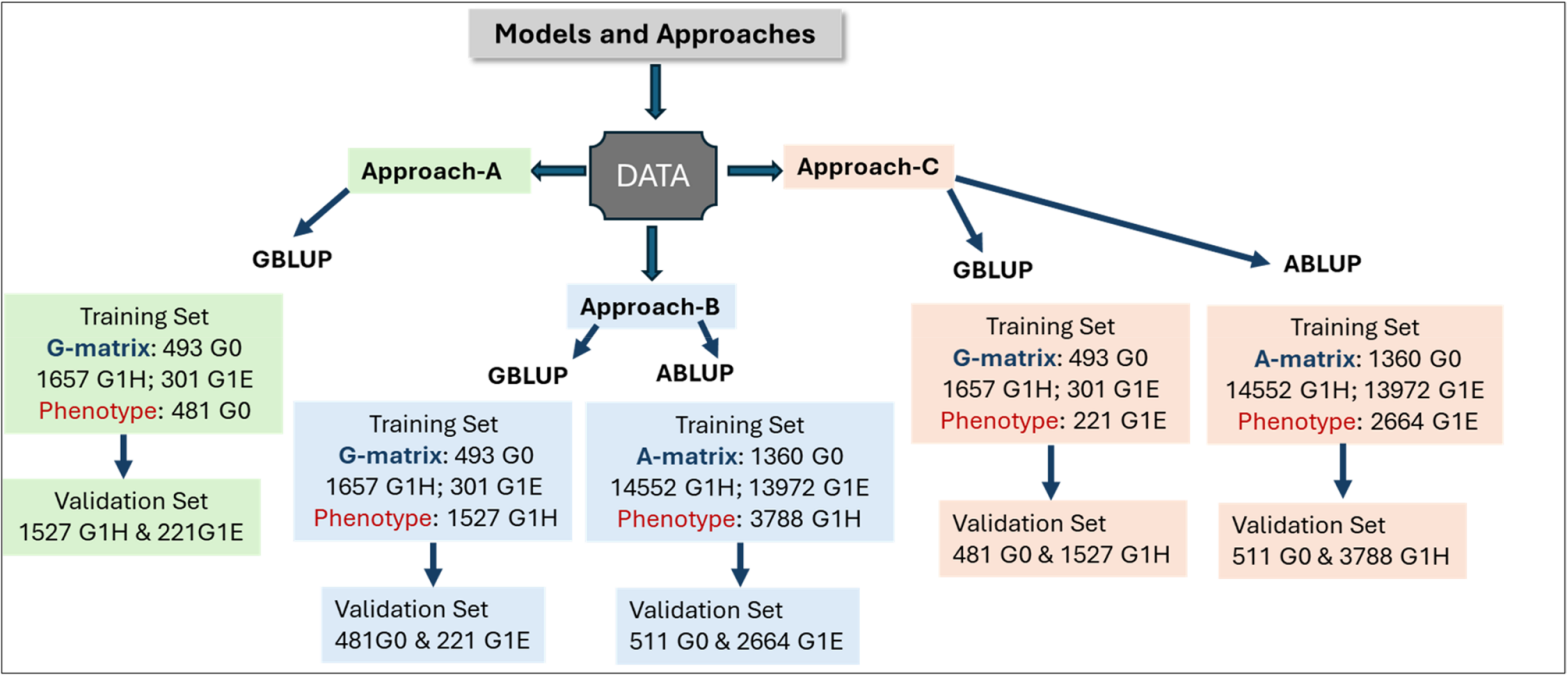
Graphical representation of cross-generation assessment comparing pedigree-based (ABLUP) and marker-based (GBLUP) models under three different approaches (Approach-A, Approach-B, and Approach-C) based on two progeny trials (G1H and G1E) and parental breeding archive clones (G0) of Norway spruce in Sweden

Genomic DNA was extracted from buds or from needles when buds were unavailable. Genotyping was performed at Rapid Genomics, USA, using exome capture sequencing, following methods similar to those described in [36]. Briefly, sequence capture was conducted using 40,018 diploid probes previously designed for *P. abies* [37]. Samples were sequenced to an average depth of 15x on an Illumina HiSeq 2500 platform. Variant calling was performed with the Genome Analysis Toolkit (GATK) HaplotypeCaller v3.6 for all samples and then all samples were jointly called. To improve the quality of called SNPs, we filtered SNPs by removing indels, keeping only bi-allelic sites, removing sites with minor allele frequency < 0.05, removing sites and individuals with more than 70% SNP missingness, and removing SNPs with an excess of heterozygotes and deviation from Hardy-Weinberg equilibrium. These filtering criteria reduced the dataset to 2,452 individuals, including 493 G0 trees and 1,958 G1 trees from 1,321 families, along with 194,831 SNPs. We used ASRgenomics package [38] in the R environment [39] for data organization and filtering of missing data. The final number of genotyped and phenotyped individuals with high-quality data retained for the analysis are presented in Table 1; Fig. 1.

**Table 1:** Number of individuals used for the ABLUP and GBLUP analysis Under three different approaches

Number of individuals	G0	G1H	G1E
Available individuals in pedigree	1360	14552	13972
Individuals with phenotypes	511	3788	2664
Individuals with genotypes	493	1657	301
Individuals with both phenotypes & genotypes	481	1527	221

G0: mother plus-trees; G1H: progenies in Höreda trial; G1E: progenies in Erikstorp trial

The discrepancies between number of phenotypes and genotypes are because not always the same individuals were selected for both genotyping and phenotyping

## Statistical analysis for pedigree-based and genome-based predictions

Prior to conducting genomic prediction analysis, a mixed linear model was applied to each trait in each progeny trial to account for environmental differences and reduce their impact on trait evaluation. The model is as follows:

2 $$\:y=X\beta\:+Wb+Zu+e$$

where $\:y$ is a vector of phenotypic observations of a single trait, $\:\beta\:\:$is a vector of fixed effects, including a grand mean, $\:b$ is a vector of post-block effects and $\:u$ is a vector of random additive genetic effect of individuals, assumed to follow a normal distribution , and $\:e$ is the residual error term, also assumed to follow a normal distribution $\:N(0,\:I{\sigma\:}_{e}^{2}$), where $\:{\sigma\:}_{A}^{2}$ and $\:{\sigma\:}_{e}^{2}$ are the additive genetic and residual variances, respectively. $\:A$ represents the additive numerator relationship matrix estimated from pedigree information, and $\:I$ is the identity matrix.

The incidence matrices $\:X$, $\:W$, and $\:Z$ correspond to the fixed effects $\:\beta\:$, post-block effects $\:b$, and random genetic effects $\:u$, respectively. The phenotypic data for each trait were adjusted by removing the variation associated with environmental design features and post-block effects for all individuals. These adjusted phenotypes ($\:y{\prime\:}$) were then used to calculate trait heritability and to develop prediction models.

Two different models were used to estimate breeding values: Genomic BLUP (GBLUP) and pedigree-based BLUP (ABLUP).

These approaches followed the framework of the mixed model:

3 $$\:{y}^{{\prime\:}}=Xb+Zu+e$$

where $\:{y}^{{\prime\:}}$ is a vector of adjusted phenotypic observations, $\:b$ is a vector of fixed effect (intercept), $\:u$ and $\:e$ are vectors of random additive genetic and residual error effects, respectively. The incidence matrices $\:X$ and $\:Z$ correspond to the fixed effects $\:b$ and random additive genetic effects $\:u$, respectively. The mixed model equations were solved to obtain estimated breeding values according to the model in Eq. (3).

It has been demonstrated that there is a strong population structure among the individuals included in this study [40]. Therefore, in the ABLUP method, the effect of population structure is incorporated as contemporary genetic groups directly within the pedigree [41]. The pedigree-derived relationship matrix (A) is used in Eq. (3) to predict estimated breeding values (EBVs). In GBLUP, the inverse of the realized genomic relationship matrix ($\:{G}^{-1}$) replaces ($\:{A}^{-1}$) to predict genomic estimated breeding values (GEBVs). Similarly to Eq. (1), the vectors of random additive effects ($\:u$) and residual effects ($\:e$) were assumed to follow normal distributions. For ABLUP, $\:u$ was assumed to follow $u\;N(0,A\sigma_u^2)$, and for GBLUP, $u\;N(0,G\sigma_u^2)$.

The G-matrix was calculated following VanRaden’s approach [42] as:

$\:G=\frac{\left(M-P\right){(M-P)}^{T}}{2\sum\:_{i=1}^{q}{p}_{i}(1-{p}_{i})}$, here $\:M$ is the allele-sharing matrix, where rows represent the total number of genotyped individuals and columns represent the total number of markers, coded as 0 for homozygous reference allele, 1 for heterozygous, and 2 for homozygous alternative allele; $\:P$ is a matrix of allele frequencies with the $\:i$-th column given by 2($\:{p}_{i}$− 0.5), where $\:{p}_{i}$ is the observed allele frequency for the $\:i$-th marker of all genotyped samples.

Narrow-sense heritability ($\:{h}^{2}$) for ABLUP and GBLUP models was calculated as:

4 $$\:{h}^{2}=\frac{{\sigma\:}_{a}^{2}}{{\sigma\:}_{a}^{2}{+\sigma\:}_{e\:}^{2}}$$

where $\:{\sigma\:}_{a}^{2}$ and $\:{\sigma\:}_{e}^{2}$ are the additive genetic and residual variances, respectively, obtained from each model.

### Model evaluation

To independently validate the efficiency of selection models, the two-generation dataset was divided into a training set (TS) comprised of both genotypic and phenotypic data for model development, and a validation set (VS), where phenotypes were predicted solely based on the model and genotypic data. Model evaluation was carried out based on predictive ability ($\:PA$), defined as the Pearson correlation$\:\:(r$) between the adjusted phenotypes and the model predicted phenotypes, i.e. $\:r=corr({y}^{{\prime\:}},\widehat{y}$), and prediction accuracy ($\:ACC$), defined as $\:\frac{PA}{\sqrt{{h}^{2}}}$, across all studied traits. For the subdivision of the dataset into TS and VS, three different approaches were used (Fig. 1).

In Approach-A, relationship matrices ($\:G$) was constructed using genotype data from all available individuals (both progeny trials and mothers), while the models were trained solely on the phenotypic data of the mother plus trees. Predictions were then evaluated for progeny trees in both Erikstorp and Höreda trials. In Approach-B, the $\:A$ and $\:G$ matrices were built using the genotype data of all individuals, but the models were trained solely on the phenotypic data from the Höreda trial. Predictions were then evaluated for trees in Erikstorp and for the mother plus trees. In Approach-C, the $\:A$ and $\:G$ matrices were built using the genotype data of all individuals, but models were trained solely on the phenotypic data from the Erikstorp trial. Predictions were then evaluated for trees in Höreda and for the mother plus trees.

## Assessment of early training and measurement method in GBLUP

The increment cores collected from mother plus trees (G0) contained up to 29 annual rings along the cambial age, with most cores comprising 21 rings. In progeny trees (G1), the cores had a maximum of 21 rings, while the majority contained 16 rings. To assess the efficiency of early training in GBLUP, the G-matrix was constructed using genotype data from both G0 and G1 trees (Approach-A). As such, the models were trained exclusively on G0 trees, using different sets of wood density measurements of the mother plus trees, while the predictions for each dataset were then validated on G1 trees from the Höreda and Erikstorp trials, using their whole-core area-weighted wood density measurements.

We trained the GBLUP model using two distinct types of datasets. The first type used accumulative area-weighted wood density (AWE-GBLUP), integrating density cumulatively from the pith (the innermost ring) to a final ring of choice. This final ring ranged from the 1 st to the maximum 21 st from pith as most wood strips from G0 trees contained reliable measurements for wood properties up to ring number 21. This approach aimed to determine at which age the genomic predictions from mothers would be most efficient for selection of their progenies given that a full radial density profile could be obtained, e.g. by analysing increment cores. The second type of datasets comprised density data obtained from single individual annual rings (SAD-GBLUP), ranging from the 1 st to the 21 st ring from pith. This approach simulated a scenario in which density measurements were collected using non-destructive, fast, and inexpensive methods, such as pilodyn [43] and Hitman [44], applied directly under the bark at a range of different timepoints during tree development.

Additionally, to check whether wood juvenility, which usually is more variable compared to juvenile wood, had an excessive influence on the results, we compared the PA from validations using only the 10 innermost rings of progeny wood cores (juvenile core) with that using all available rings.

## Results

### Quantitative-genetic parameters and relatedness

**Fig. 2:**
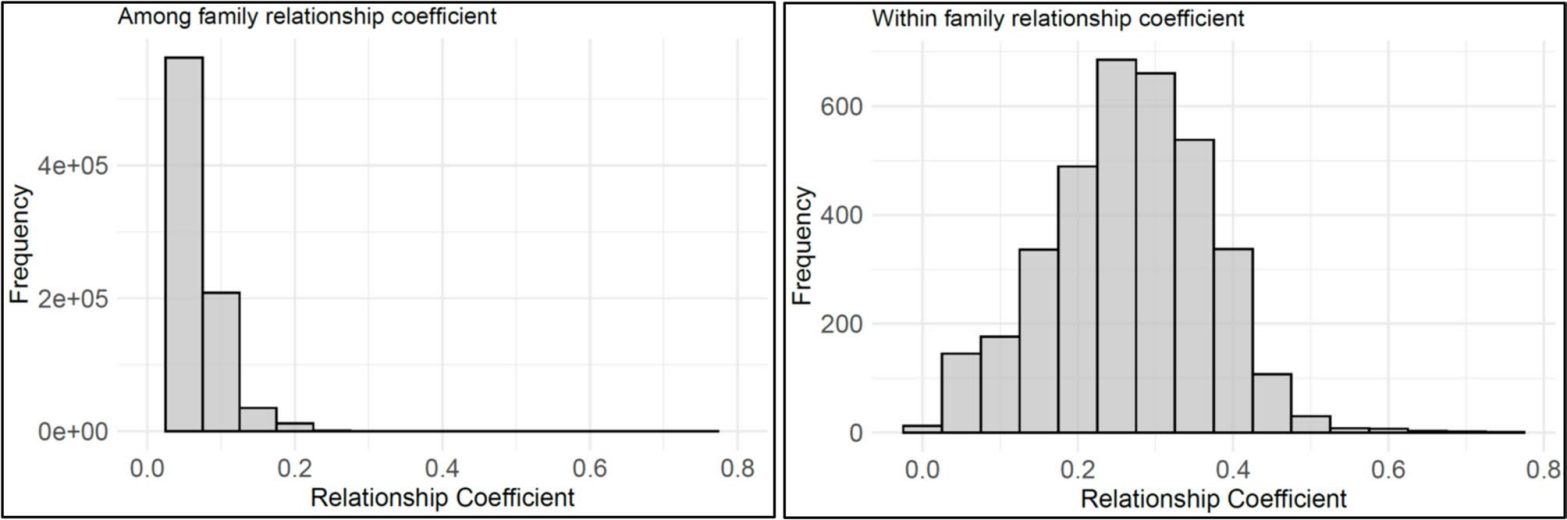
Histogram of genomic pairwise relationship coefficients between individuals across (left panel) and within (right panel) Norway spruce open-pollinated (OP) families

The distribution of genomic pairwise relationship coefficients within and among Norway spruce open-pollinated (OP) families used in this study is shown in Fig. 2. The left panel illustrates the distribution of relationship coefficients among families, with most values clustering around 0.00 (mean = 0.05), indicating no relationship. The right panel presents the distribution of relationship coefficients within families, with most values clustering around the expected 0.25 (mean = 0.26). However, some individuals deviate from 0.25, suggesting an imperfect half-sib family structure.

The genetic parameter estimates for various wood traits under ABLUP and GBLUP models reveal notable patterns in additive genetic variance ($\:{\sigma\:}_{A}^{2}$), residual variance$\:\:({\sigma\:}_{e}^{2}$), and narrow-sense heritability estimates ($\:{h}^{2}$) (Table 2). Approach-A, yielded higher $\:{\sigma\:}_{A}^{2\:}$ and, therefore, higher $\:{h}^{2}$ estimates for density (DENS) and its three components, including earlywood (EWD), transitionwood (TWD), and latewood density (LWDENS), compared to tracheid wall thickness (TWTH) and tracheid coarseness (TC). These estimates were mostly zero for ring width (RWT), modulus of elasticity (MOE), and microfibril angle (MFA).

**Table 2:** Additive variance $(\sigma_A^2)$, Residual variance $(\sigma_e^2)$, and Narrow-sense heritability $(h^2)$ with their standard errors (±SE), from ABLUP and GBLUP models for nine different wood properties measured in Norway spruce

Trait	Approach	Trained data	Additive variance $(\sigma_A^2)$		Residual variance $(\sigma_e^2)$		Narrow-sense heritability $(h^2)$	
			ABLUP	GBLUP	ABLUP	GBLUP	ABLUP	GBLUP
DENS	A	G0	NA	612.284 (356.346)	NA	978.316 (458.689)	NA	0.384 (0.246)
	B	G1H	1041.527 (114.337)	380.470 (108.856)	397.987 (100.456)	916.750 (137.573)	0.723 (0.072)	0.293 (0.089)
	C	G1E	664.338 (96.695)	703.957 (291.097)	553.442 (86.447)	137.162 (314.406)	0.545 (0.073)	0.836 (0.367)
EWDENS	A	G0	NA	168.308 (95.332)	NA	302.923 (123.240)	NA	0.357 (0.221)
	B	G1H	428.212 (47.818)	181.418 (47.530)	159.442 (42.013)	352.030 (59.278)	0.728 (0.073)	0.340 (0.095)
	C	G1E	257.284 (39.217)	181.688 (114.837)	244.056 (35.370)	195.875 (131.263)	0.513 (0.073)	0.481 (0.321)
TWDENS	A	G0	NA	631.197 (286.197)	NA	568.799 (362.806)	NA	0.525 (0.270)
	B	G1H	676.495 (91.330)	334.379 (86.470)	559.101 (80.274)	753.093 (109.147)	0.547 (0.066)	0.307 (0.084)
	C	G1E	632.409 ( 98.203)	470.343 (284.418)	645.384 (89.029)	485.796 (324.432)	0.494 (0.072)	0.491 (0.314)
LWDENS	A	G0	NA	1425.027 (560.087)	NA	1069.156 (705.878)	NA	0.571 (0.255)
	B	G1H	928.969(161.008)	570.011 (159.936)	1578.36 (153.236)	1653.805 (205.923)	0.370 (0.062)	0.256 (0.075)
	C	G1E	1501.219 (267.673)	866.382 (650.081)	1278.462 (196.591)	1624.849 (764.519)	0.540 (0.073)	0.347 (0.273)
RWT	A	G0	NA	0.002 (0.097)	NA	1.345 (0.157)	NA	0.001 (0.072)
	B	G1H	0.275 (0.036)	0.098 (0.035)	0.236 (0.032)	0.453 (0.047)	0.537 (0.065)	0.178 (0.067)
	C	G1E	0.154 (0.036)	0.165 (0.139)	0.392 (0.035)	0.395 (0.166)	0.282 (0.064)	0.294 (0.259)
MOE	A	G0	NA	3.90e-07 (NA)	NA	3.992 (0.917)	NA	0.00
	B	G1H	1.593 (0.291)	0.711 (0.289)	3.010 (0.279)	3.838 (0.388)	0.346 (0.061)	0.156 (0.065)
	C	G1E	1.468 (0.311)	0.323 (0.504)	3.051 (0.298)	3.349 (0.686)	0.324 (0.066)	0.088 (0.139)
MFA	A	G0	NA	0.335 (1.664)	NA	20.190 (2.572)	NA	0.016 (0.081)
	B	G1H	2.970 (0.999)	1.813 (1.070)	15.088 (1.022)	17.331 (1.496)	0.164 (0.054)	0.095 (0.06)
	C	G1E	3.687 (1.298)	3.622 (4.063)	17.622 (1.328)	18.217 (5.114)	0.173 (0.060)	0.165 (0.191)
TWTH	A	G0	NA	0.009 (0.006)	NA	0.024 (0.008)	NA	0.288 (0.206)
	B	G1H	0.019 (0.002)	0.007 (0.002)	0.013 (0.002)	0.022 (0.003)	0.585 (0.068)	0.25 (0.08)
	C	G1E	0.013 (0.002)	0.013 (0.005)	0.011 (0.001)	0.004 (0.006)	0.501 (0.071)	0.773 (0.328)
TC	A	G0	NA	100.778 (138.430)	NA	889.317 (189.915)	NA	0.101 (0.144)
	B	G1H	397.691 (64.486)	134.075 (62.047)	581.305 (60.742)	743.338 (82.438)	0.406 (0.063)	0.153 (0.073)
	C	G1E	315.402 (53.965)	392.264 (162.005)	398.781 (48.623)	191.819 (178.172)	0.441 (0.070)	0.671 (0.292)

G0: mother plus-trees; G1H: progenies in Höreda trial; G1E: progenies in Erikstorp trial

*DENS: W*ood density, *EW *Earlywood, *TW *Transition wood, *LW *Latewood, *RWT *Ring width, *MOE *Modulus of elasticity, *MFA *Microfibril angle, *TWTH *Tracheid wall thickness, *TC *Tracheid coarseness

The greatest observed difference between the trained models were the substantial reduction in $\:{\sigma\:}_{A}^{2}$ estimate, which was followed by a decrease in $\:{h}^{2}$, when the GBLUP model was used, compared to the ABLUP model. This was particularly noticeable for density-related properties. For instance, when trained on G1H data (Approach-B), the$\:{\sigma\:}_{A}^{2\:}$ estimates from GBLUP were approximately 63%, 57%, 50%, and 38% lower than those from the ABLUP model for DENS, EWDENS, TWDENS, and LWDENS, respectively. When trained on G1E data (Approach-C), the reductions for EWDENS, TWDENS, and LWDENS were 29%, 25%, and 42%, respectively, while for DENS, GBLUP yielded a $\:{\sigma\:}_{A}^{2\:}$estimate nearly the same as ABLUP.

Similarly, the $\:{\sigma\:}_{A}^{2\:}$estimates from the GBLUP model were about 64%, 55%, 39%, 63%, and 66% lower than those from ABLUP for RWT, MOE, MFA, TWTH, and TC under Approach-B. In contrast, under Approach-C, the $\:{\sigma\:}_{A}^{2\:}$estimates from GBLUP were about 7% and 24% higher than ABLUP for RWT and TC, respectively. For MFA and TWTH, the estimates were mostly equal, whereas for MOE, GBLUP yielded a much lower estimate.

As expected, the $\:{h}^{2}$ estimates followed a similar pattern to the$\:\:{\sigma\:}_{A}^{2}$ estimates. In general, $\:{h}^{2}$ estimates from ABLUP were higher than those from GBLUP under Approach-B. However, under Approach-C, $\:{h}^{2}$ estimates from GBLUP were higher or similar to ABLUP, except for MOE.

Moreover, the$\:\:{\sigma\:}_{e}^{2}$ estimates from ABLUP were generally lower than those from GBLUP under Approach-B. In contrast, under Approach-C, GBLUP yielded mostly lower $\:{\sigma\:}_{e}^{2}$ estimates than ABLUP (Table 2).

### Evaluation of the models’ performances

**Fig. 3:**
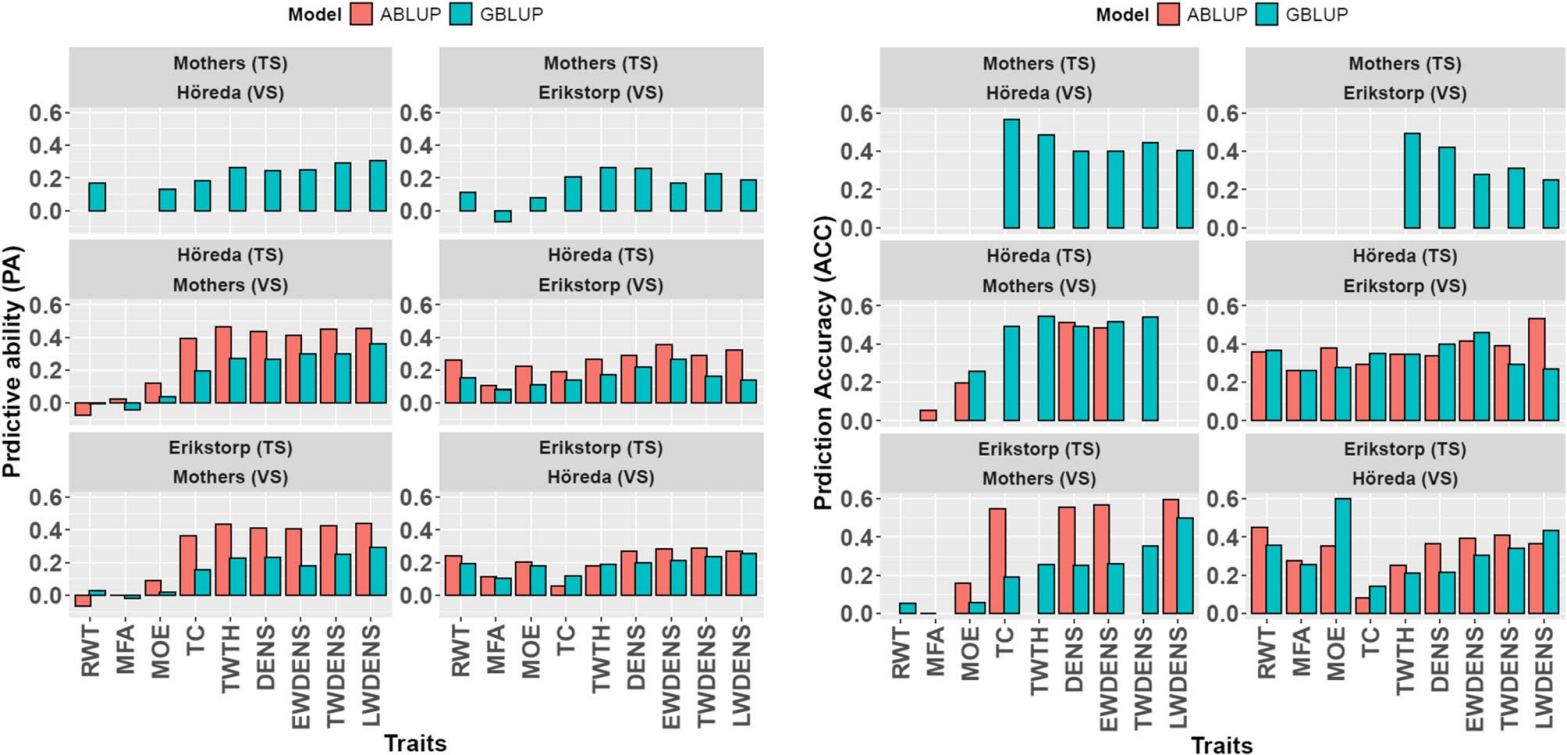
Predictive ability (PA) and prediction accuracy (ACC) of ABLUP and GBLUP models trained and validated under three different approaches for nine different wood properties in two generations of Norway spruce. In Approach-A, models were trained using phenotype data from mother (G0) trees and validated on progeny (G1) in Höreda (G1H) and Erikstorp (G1E). In Approach-B, models were trained using phenotype data from G1H and validated on G0 and G1E. In Approach-C, models were trained using phenotype data from G1E and validated on G0 and G1H. TS: training set; VS: Validation set.

The performances of ABLUP and GBLUP models were evaluated based on their predictive ability (PA) and prediction accuracy (ACC) for various wood properties, as shown in Fig. 3 and Table S1. Overall, RWT, MOE, and MFA exhibited lower PA and ACC compared to wood density and its components (EWDENS, TWDENS, LWDENS), TWTH, and TC across all models.

Under Approach-A, the PA values for density-related properties, TWTH, and TC based on GBLUP ranged from 0.18 to 0.31 when validated on G1H and from 0.17 to 0.26 when validated on G1E. In contrast, the PA values for RWT, MOE, and MFA based on GBLUP was substantially lower, ranging from − 0.11 to 0.17 when validated on G1H and from − 0.07 to 0.11 when validated on G1E.

When comparing ABLUP and GBLUP under Approach-B, with validation on G0 trees, values of PA ranged from 0.40 to 0.46 for ABLUP and from 0.19 to 0.36 for GBLUP in density-related traits, TWTH and TC. A similar pattern was observed with models trained under Approach-C and validated on G0 trees, where PA ranged from 0.36 to 0.44 for ABLUP, and from 0.16 to 0.29 for GBLUP. Under these approaches and validation on G0 trees, PA values for RWT, MOE, and MFA, ranged from − 0.08 to 0.26 across ABLUP and GBLUP models.

When models were trained under Approach-B and validated on G1E individuals, PA values for density-related properties, TWTH, and TC ranged from 0.19 to 0.36 for ABLUP and from 0.14 to 0.27 for GBLUP. For RWT, MOE, and MFA, under the same approach, PA values were lower, ranging from 0.08 to 0.26 across both models.

Similarly, when models were trained under Approach-C and validated on G1H individuals, PA values for density-related properties, TWTH, and TC ranged from 0.05 to 0.29 for ABLUP and from 0.12 to 0.26 for GBLUP. For RWT, MOE, and MFA in this scenario, PA values ranged from 0.12 to 0.24 across the models.

The ACC patterns were generally similar to those observed for PA, with MFA, MOE, and RWT showing lower ACC values across models and approaches. For instance, when the GBLUP model was trained under Approach-A and validated on G1H and G1E trees, the ACC values for RWT, MOE, and MFA were nearly zero or inestimable. In contrast, for density-related properties, TWTH, and TC, the same model yielded ACC values ranging from 0.40 to 0.57 when validated on G1H and from 0.25 to 0.66 when validated on G1E.

When models were trained under Approach-B and validated on G0, ACC values for density-related properties, TWTH, and TC ranged from 0.48 to 0.75 for ABLUP and from 0.49 to 0.71 for GBLUP. Under Approach-B and validation on G1Eindividuals, ACC values for such traits ranged from 0.30 to 0.53 for ABLUP and from 0.27 to 0.46 for GBLUP.

For models trained under Approach-C and validated on G0, ACC ranges for density-related traits, TWTH, and TC were 0.55 to 0.61 for ABLUP and 0.19 to 0.50 for the GBLUP. However, when validated for G1H individuals, ABLUP model yielded values from 0.08 to 0.41 and GBLUP model yielded values from 0.14 to 0.44.

### Assessment of early training and phenotyping methods in GBLUP

**Fig. 4:**
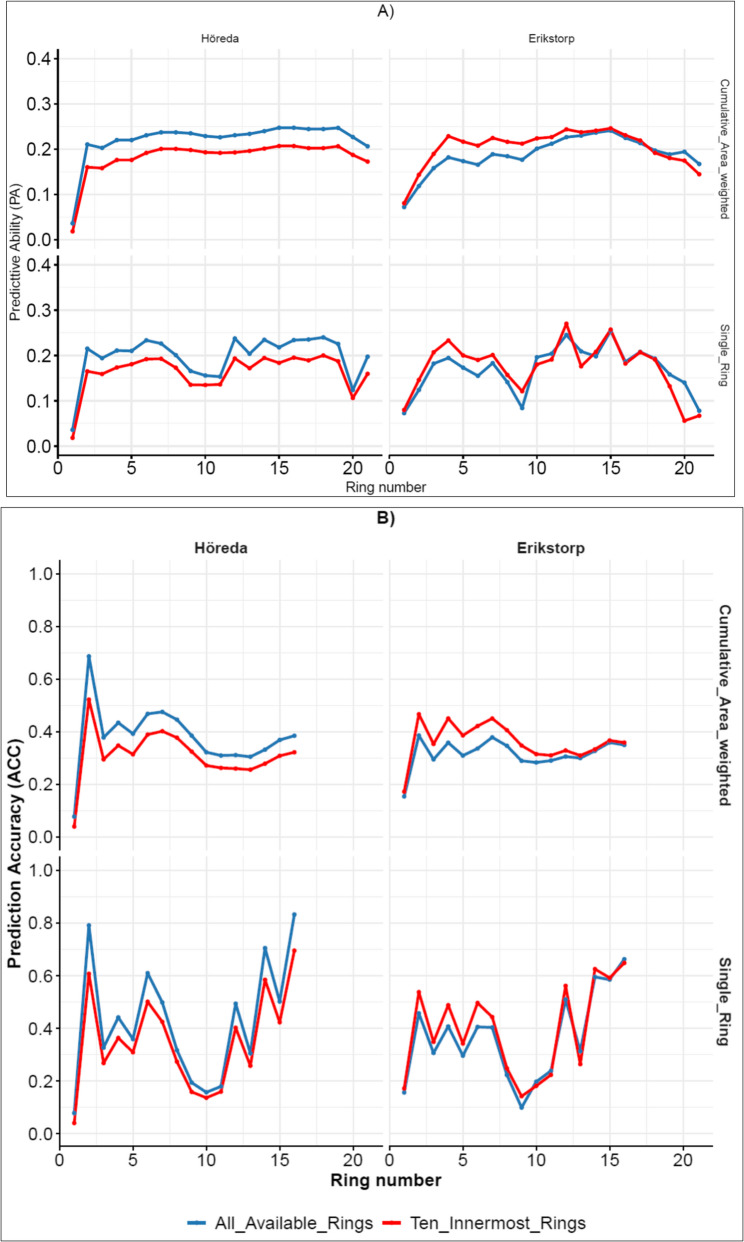
Predictive ability (PA) (**A**) and prediction accuracy (ACC) (**B**) for wood density of GBLUP models under Approach-A trained using (1) cumulative area-weighted (top panel) and (2) single annual-ring direct (bottom panel) wood density measurements from individual rings 1 to ring 21 of mother plus trees (G0) and validated using wood density data from the juvenile portion (innermost 10 annual rings, red line) and whole core data (all available rings, blue line) of progeny in Höreda and Erikstorp

Figure 4 presents the PA (A) and ACC (B) of GBLUP models trained under Approach-A using wood density (DENS) measurements from rings 1 to 21 of G0 trees. Two training methods were used: (1) cumulative area-weighted estimates (AWE-GBLUP), reflecting evaluations based on the whole radial profile, and (2) single annual-ring direct DENS measurements (SAD-GBLUP), reflecting non-destructive sampling under the bark at a specific timepoint. These models were then validated using DENS data from (1) the juvenile portion (innermost 10 annual rings) and (2) whole core data (all available rings) of progeny in Höreda (G1H) and Erikstorp (G1E).

Across both trials, PA based on both validation alternatives (juvenile wood or all available rings) increased from the pith, peaked around rings 12–18, and then declined toward the bark.

In Höreda, PA based on AWE-GBLUP reached a maximum value of 0.25 when validated using all available rings of progenies and a maximum value of 0.21 when validated using only the innermost 10 annual-rings, both at ring number 16. In Erikstorp, PA based on AWE-GBLUP peaked at approximately 0.25 at ring number 15 for both validation methods.

When trained using SAD-GBLUP, PA values for both validation methods in both trials fluctuated along the cambial age. Initially, PA increased from the pith to ring number 7, then declined significantly between rings 9 and 11, before increasing again and stabilizing toward the bark.

Similar to the trend observed for PA, the ACC values of SAD-GBLUP model exhibited greater fluctuations along the cambial age compared to the AWE-GBLUP model. When validated on G1H, ACC values reached their lowest levels between annual rings 9 and 11, aligned with the lowest PA values, whereas $\:{h}^{2}$ estimates (Fig. S1 and Table S2) were highest at these ages. As the PA of the models declined beyond ring 16, the corresponding ACC values became increasingly unstable and potentially unreliable. Therefore, ACC values are presented up to ring 16 in Fig. 4.

## Discussion

To the best of our knowledge, this is the first study to investigate genomic prediction in Norway spruce using a two-generation dataset that includes quality and growth traits assessed from wood increment cores in two different environments. Our results provide practical insights for the operational deployment of genomic selection (GS) in conifer breeding, particularly for economically important wood quality traits like density. We explored the efficiency of forward and backward GS across two generations and environments using three different assessment approaches. We specifically evaluated the performance of the GBLUP model under Approach-A across multiple cambial ages. The model was independently validated using wood density data representing both the early and later growth phases of progeny in Höreda (G1H) and Erikstorp (G1E). The main objective was to identify the optimal selection age for GBLUP and to evaluate if wood juvenility influences the outcomes of GS.

Additionally, we compared the GBLUP models trained using both cumulative area-weighted density (AWE-GBLUP) and single annual-ring density measurements (SAD-GBLUP) from the parental mother (G0) trees. The motivation was to determine whether the added cost and effort of accumulative measurements, which provide a comprehensive radial history of wood development, are justified, or if the more practical direct methods, which primarily assess a limited number of rings near the bark, suffice for accurate breeding decisions. Our findings underscore the importance of developing context-specific models to enhance the accuracy and reliability of genomic prediction in forest tree breeding.

Various statistical methods are available for predicting genetic values, broadly classified into two groups: those that estimate individual marker effects (e.g., Bayesian shrinkage, ridge regression, Bayesian LASSO) [45] and those that utilize genomic relationships among individuals derived from the markers (e.g., GBLUP) [46]. While empirical and simulation studies in forestry suggest that the choice of statistical method has a small impact on the efficiency of GS [4, 22, 23, 47], GBLUP is generally preferred for routine genomic evaluations due to its computational efficiency and similarity to the traditional pedigree-based BLUP model [22, 23, 48]. Furthermore, unlike traits such as disease resistance, which may be controlled by a few large-effect genes and are better predicted using Bayesian-based methods [49], key forestry traits such as growth and wood properties exhibit polygenic inheritance, controlled by many small-effect loci, making GBLUP models well-suited for their prediction [23]. Therefore, to evaluate the efficiency of GS for growth and wood properties, we primarily focused on GBLUP and compared it with pedigree-based (ABLUP) approaches.

### Quantitative-genetic parameters and relatedness

It is well-recognized that reliable estimates of additive genetic variance ($\:{\sigma\:}_{A}^{2}$) are critical for the success of genetic improvement programs [40]. Nevertheless, $\:{\sigma\:}_{A}^{2}\:$estimates derived from offspring of open-pollinated (OP) families might be inflated because the assumption of ‘half-sibling’ relationships is rarely met. Moreover, OP family mating schemes face challenges in disentangling additive from non-additive genetic effects due to shallow pedigrees and a lack of connectedness among the tested families [50–52].

In our study, we observed a substantial reduction in $\:{\sigma\:}_{A}^{2}$, and consequently, a decrease in narrow-sense heritability estimate ($\:{h}^{2}$) when the GBLUP model was applied, compared to the ABLUP model, particularly for density-related traits. This reduction is not necessarily indicative of lower model performance but rather reflects the fact that GBLUP captures realized genomic relationships using SNP data. Notably, the estimated genomic pairwise relationships among individuals within families often deviated from the expected coefficient of relatedness of 0.25 for half-siblings, while relationships among individuals from unrelated families remained close to the expected value of 0.0 (Fig. 2). Such findings suggest that discrepancies in the expected pedigrees, and therefore in the pairwise relationships, were effectively corrected using SNP data. Additionally, the dense SNP data likely captured hidden genetic variation among unknown fathers and possibly also Mendelian sampling/segregation within families, allowing genetic variance estimates to reflect the true genomic proportions among half-siblings.

However, it is worth noting that the number of individuals used in ABLUP was not equal to that used in GBLUP. To ensure a fair comparison between the two models, analyses should ideally be based on the same set of individuals. The observed differences may, therefore, partly reflect this imbalance. Nevertheless, we performed an additional analysis using a subset of individuals common to both models for density, and the results were consistent with those obtained using the full ABLUP dataset (data not shown). That said, a direct comparison between ABLUP and GBLUP is not the main objective of this study; rather, our main objective was to assess the predictive performance of models using the existing data.

### Evaluation of the models’ performances

The evaluation of GS efficiency in forestry, and many other crops, has primarily relied on cross-validation schemes, often by splitting the same generation for both model training and validation. While this approach provides valuable insights into GS performance within a specific context, its reliability is diminished when applied to multigenerational breeding. This is due to the decrease in relatedness between the training set (TS) and validation set (VS) [53] as well as changes in linkage disequilibrium (LD), caused by genetic recombination, selection, and drift [54, 55]. Although limited, a few studies have explored the utility of GS across generations, including those in maritime pine (*Pinus pinaster* Ait.) [4] and *E. grandis* [56]. In the latter study, the effectiveness of the GS model was further evaluated by measuring the realized PA, which was obtained by comparing the genomic estimated breeding values (GEBVs) with actual phenotypic data for volume growth, wood density, and pulp yield across four generations. The authors concluded that GS is more efficient for predicting wood quality traits but remains challenging for predicting growth traits.

As breeding programs advance in their implementation of GS, the need for rigorous validation becomes increasingly critical. This includes predicting progeny performance for forward selection, predicting parental performance for backward selection, and assessing genomic performance across trials. In this study, we evaluated the accuracies of ABLUP and GBLUP predictions using an independent validation method, where no individuals or environments were shared between TS and VS, ensuring a true validation of evaluations. In general, and as expected, Predictive ability (PA) and prediction accuracy (AC) followed a similar trend to their corresponding narrow-sense heritability estimates ($\:{h}^{2}$). PA and ACC for wood properties were significantly higher than for ring width (RWT), modulus of elasticity (MOE), and microfibril angle (MFA), traits associated with lower$\:\:{h}^{2}$. These results align well with previous studies comparing the genetic control and prediction efficiencies of growth and wood properties in Norway spruce [30, 31, 57] and some other species [56]. Such pattern is consistent with the general findings in forest trees, where adaptive traits, such as growth-related properties, exhibit complex inheritance patterns and often have lower heritability due to strong environmental influences and polygenic control [58]. In contrast, wood properties, may be influenced by genes with alleles of larger effect or by genes in stronger linkage disequilibrium (LD) with markers, giving them greater power to explain phenotypic variation [59].

Regarding MFA and MOE, it is well-documented that the estimated genetic parameters of these traits often exhibit high standard errors. This is most likely due to significant within- and among-tree variations [60], as well as the challenges in obtaining precise measurements for these traits [61]. As such, ACC for RWT, MOE, and MFA using forward GBLUP, backward ABLUP and GBLUP models, was mostly inestimable due to the very low or nearly zero estimates of PA and $\:{h}^{2}$ for these traits. Despite this, the ACC values of RWT, MFA, and MOE were significantly higher when the ABLUP and GBLUP models were used to predict the performance of progenies in a separate trial. We speculate that this observation results from the model leveraging the shared genetic structure among progenies within the same generation, particularly given the low G × E interaction levels observed for these traits across the two trials [29].

Theoretically, genomic prediction of non-phenotyped genotypes based on the GBLUP approach is strongly dependent on the relationship between the TS and the VS. In the context of pedigree inheritance, a parent contributes 50% of its genetic material to its offspring, whereas half-sib individuals, exhibit an expected genetic relatedness of 25%. Based on this, higher ACC would be expected for cross-generation predictions compared to within- generation predictions of half-sib individuals. This assumption, however, was only valid for high-heritability traits in our study, particularly for backward selection predictions, and especially when a larger and diverse set of individuals were included in the TS (Approach-B). This finding further supports the idea that for high-heritability traits, the haplotype structures and genetic relatedness responsible for prediction accuracy are preserved and remain more stable across the breeding cycles [62].

It is worthwhile to mention that dominance and epistatic effects contribute significantly to the growth properties of Norway spruce [63–65]. Although GBLUP provides accurate predictions of breeding values, it estimates additive genetic effects unless specifically modified. Since forward GBLUP models, which largely rely only on parental phenotypes, do not capture non-additive and environmental variances, their effectiveness in prediction of growth-related properties may be limited in species like Norway spruce, where both non-additive and epigenetic effects [66] play a crucial role.

### Assessment of early training and phenotyping methods for density using GBLUP

Across both trials, PA based on both validation alternatives (juvenile vs. all available rings) increased from the pith, peaked around rings 15–16, and then declined toward the bark. This pattern may be attributed to the fact that most G1 individuals have up to 16 annual rings, with the number of individuals decreasing beyond this age toward the bark. In most cases, PA from whole-core validation was slightly higher than, or comparable to, that from juvenile-ring validation. This indicates that selection based on early assessed wood density in G0 parents can serve as an effective training set for predicting performance in their offspring, regardless of age. This finding is further supported by the strong age–age correlations previously reported for this trait in these trials [29]. Similarly, both training alternatives (single annual-ring direct measurements (SAD-GBLUP) and cumulative whole core measurements (AWE-GBLUP)) resulted in comparable PA and ACC values. This indicates that standing tree-based measurements, without the need for deep coring into the stem, can serve as a cost-effective alternative for model training, consistent with previous findings in Norway spruce [30]. However, SAD-GBLUP introduced greater variability along the cambial age, as this method focuses on a specific tree ring where wood density fluctuates between earlywood and latewood. In contrast, AWE-GBLUP reduces this variability by integrating measurements across multiple rings. Despite these differences, both training methods exhibited a similar trend, with PA and ACC decreasing between rings 7 and 11. This decline may reflect variability in wood development as trees transition from juvenile to mature wood [67]. For wood density, the transition is typically reported to occur between cambial ages 8 and 12 [68]. Additionally, this trend might be influenced by environmental factors, such as climatic fluctuations, which could have affected wood formation and contributed to increased phenotypic variability at these ages.

## Conclusions

This study represents the first genomic selection (GS) analysis in Norway spruce using a two-generation dataset incorporating secondary growth and wood quality traits assessed across two environments. Our findings highlight the potential of GBLUP-based models for effective forward and backward selection, especially for high-heritability traits such as wood density, based entirely on true validation schemes. Notably, direct measurement approaches provided comparable prediction accuracies to whole-core approaches, supporting the use of more practical and cost-effective phenotyping methods in operational breeding programs. However, certain limitations should be acknowledged. Although a direct comparison between the performance of ABLUP and GBLUP was not the primary objective of this study, some of the observed differences may partly reflect the unequal number of individuals used in each model. Additionally, lower accuracy for traits with low heritability likely reflects the influence of non-additive genetic effects and/or the need for a higher number of individuals per family. Despite these constraints, our findings emphasize the need for trait- and context-specific GS strategies in conifer breeding. Future efforts should aim to expand training populations, incorporate non-additive genetic effects, and validate model performance across cambial ages while accounting for climatic variability during the corresponding growth years. Overall, this study offers a valuable foundation for implementing GS in Norway spruce breeding programs.

## Supplementary Information

Supplementary Material 1.

Supplementary Material 2.

Supplementary Material 3.

## Data Availability

The exome capture raw reads and the RNA-seq data have been deposited in NCBI’s sequence read archive (SRA) under accession number (PRJNA731384). The phenotypic/genotypic dataset used and/or analysed during the current study are available from the corresponding author on reasonable request.

## Abbreviations

GS: Genomic selection
MAS: Marker assisted selection
QTL: Quantitative trait loci
TS: Training set, VS: validation set
GBLUP: Genomic based unbiased linear model
ABLUP: Pedigree based unbiased linear model
LD: Linkage disequilibrium
PA: Predictive ability
ACC: Prediction accuracy
G×E: Penotype by environment
Skogforsk: Forestry Research Institute of Sweden
RWT: Ring width
MOE: Modulus of elasticity
MFA: Microfibril angle
DENS: Wood density
EWDENS: Earlywood density
TWDENS: Transition wood density
LWDENS: Latewood density
TWTH: Tracheid wall thickness
TC: Tracheid coarseness
AWE: Area-weighted
AWE-GBLUP: Area-weighted GBLUP
SAD-GBLUP: Single annual ring direct GBLUP
OP: Open-pollinated
GATK: Genome analysis toolkit
G0: Mother plus trees
G1H: Progenies in Höreda trial
G1E: Progenies in Erikstorp trial
h^2^: Narrow-sense heritability estimate
