# Supplementary figures and images for: Cross-generational genomic prediction of Norway spruce (Picea abies) wood properties: an evaluation using independent validation
Source: BMC Genomics. 2025 Jul 21;26:680. doi: 10.1186/s12864-025-11861-x (PMC12278512; doi:10.1186/s12864-025-11861-x)

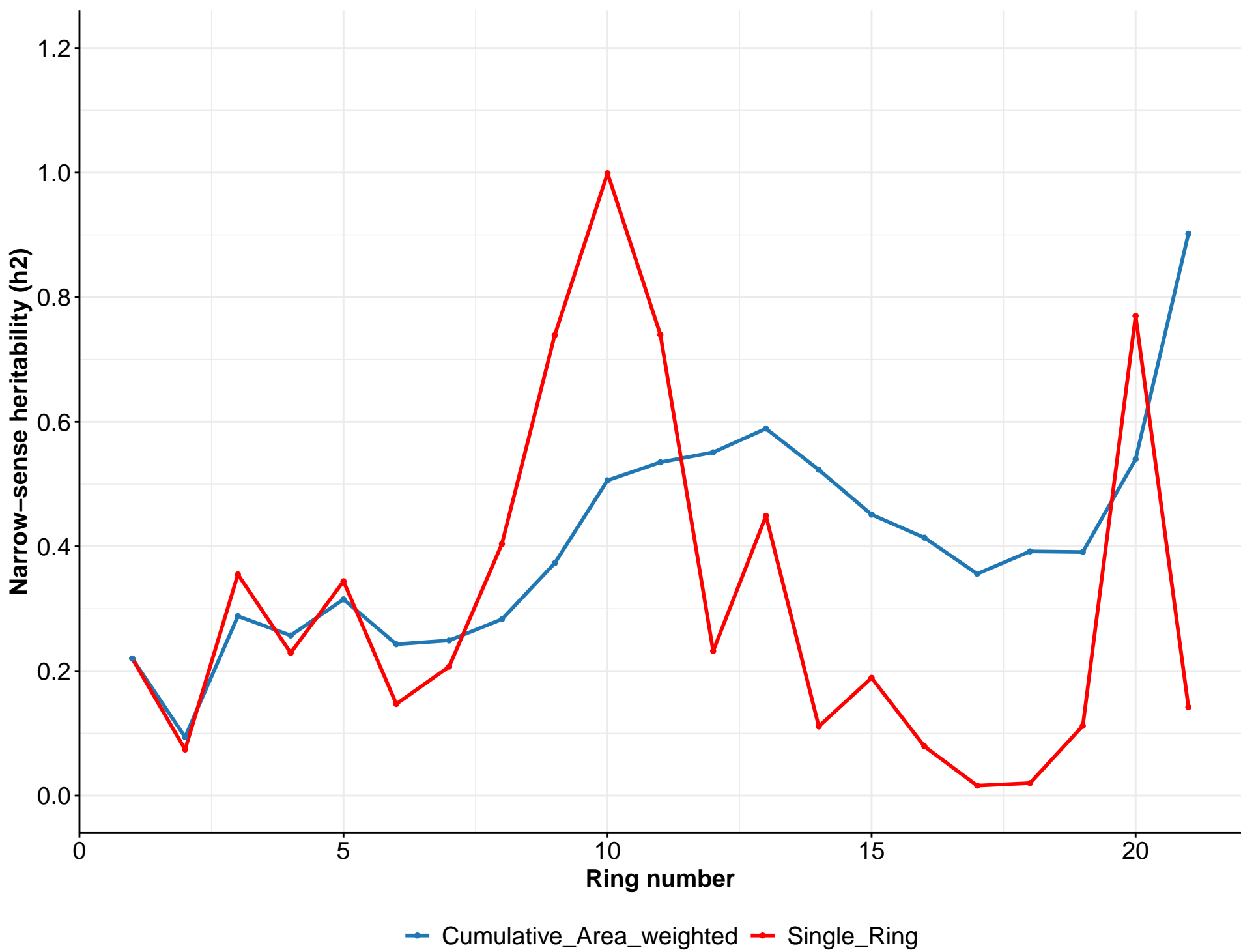

Supplement: Supplementary file 3 — Supplementary Material 3. [file 12864_2025_11861_MOESM3_ESM.pdf]
